# Indifferent, Affectionate, or Deceitful: Lifestyles and Secretomes of Fungi

**DOI:** 10.1371/journal.ppat.1002515

**Published:** 2012-03-01

**Authors:** Rohan G. T. Lowe, Barbara J. Howlett

**Affiliations:** School of Botany, The University of Melbourne, Victoria, Australia; Duke University Medical Center, United States of America

## Introduction

Fungi occupy a myriad of niches. They can be free-living (indifferent) as saprophytes recycling nutrients in the natural environment and/or have a range of relationships (affectionate and deceitful) with insect, animal, or plant hosts. Interactions with plants can be a continuum and range from obligate biotrophy where fungi cannot be cultured outside living hosts to necrotrophy where fungi kill and live on released nutrients. Biotrophic fungi need to avoid or suppress defence responses. They include symbionts, which confer a benefit to the host, and pathogens, which can cause devastating diseases such as stem rust, which threatens production of wheat worldwide [1]. Mycorrhizae colonise roots of >80% of land plants and are symbiotic, increasing nitrogen and phosphorus uptake from the soil, while feeding on sugars from the host photosynthate. Secreted proteins are on the front line of host–fungal interactions, and a particular class, effectors, is a hot topic. Here, we examine a range of fungi and consider their complement of secreted proteins (secretome) and roles of effectors in fungal lifestyles.

## For Some Fungi, There Is a Relationship between Lifestyle and Secretome Size

**Figure 1:**
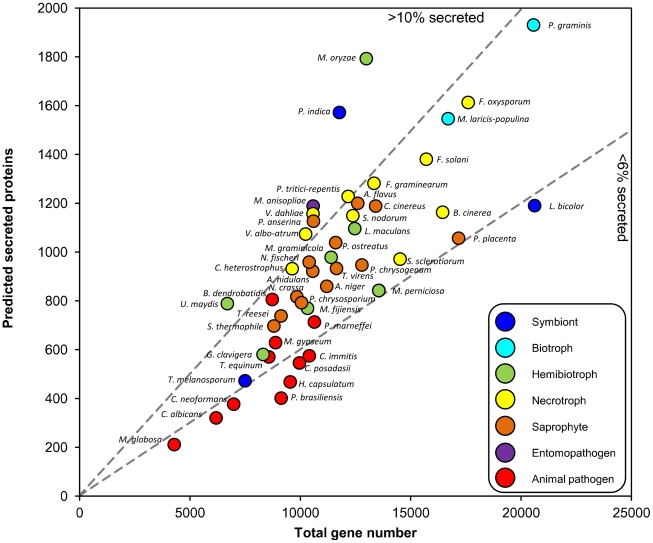
Relationship between predicted secreted protein number and total gene content of fungi. Data are from [3], or by applying SignalP to genome releases (indicated by *). Dashed lines discriminate between fungi with high (>10) or low (<6) % secreted proteins. Animal pathogens: *Batrachochytrium dendrobatidis, Candida albicans, Coccidioides immitis, C. posadasii, Cryptococcus neoformans, Histoplasma capsulatum, Malassezia globosa, Microsporum gypseum, Paracoccidioides brasiliensis, Penicillium marneffei, Trichophyton equinum*. Hemibiotrophs: Grosmania clavigera*, Leptosphaeria maculans* [14], Magnaporthe oryzae*, Mycosphaerella fijiensis, M. graminicola, Moniliophthora perniciosa, Ustilago maydis. Entomopathogen: Metarhizium anisopliae* [4]. Necrotrophs: Botrytis cinerea, Cochliobolus heterostrophus, Fusarium graminearum, F. oxysporum, F. solani, Pyrenophora tritici-repentis, Stagonospora nodorum*, Sclerotinia sclerotiorum, Verticillium albo-atrum, V. dahliae. Biotrophs: Melampsora laricis-populina* [15], *Puccinia graminis f.sp. tritici*. Saprophytes: *Aspergillus flavus, A. nidulans, A. niger, Coprinus cinereus, Neurospora crassa, Neosartorya fischeri, Podospora anserina, Penicillium chrysogenum, Phanerochaete chrysosporium, Pleurotus ostreatus, Postia placenta, Sporotrichum thermophile, Trichoderma reesei, T. virens*. Symbionts: Laccaria bicolor, Piriformospora indica* [3], Tuber melanosporum* [16].

The Fungal Secretome Database (FSD) [2] predicts secreted proteins using SignalP, which identifies secretion signal peptides within proteins. We applied SignalP to several recently completed genomes and examined whether the ratio of secretome size to total gene number reflects the predominant lifestyles (Figure 1). The total gene number ranges from 4,000 to 20,000, and the proportion of secreted proteins from 4% to 14%. Fungi with biphasic lifestyles have a large proportion of secreted proteins. These include the hemibiotrophic rice blast fungus *Magnaporthe oryzae*, the corn smut fungus *Ustilago maydis*, and *Piriformospora indica*, which colonizes dead roots saprophytically and live roots as a biotrophic symbiont [3]. Its biphasic lifestyle is reflected in its transcriptome; many genes induced during growth on living roots are similar to those of the symbiont *Laccaria bicolor*, whereas genes induced during saprophytic growth are similar to those of the saprophyte *Coprinus cinereus*. The insect pathogens *Metarhizium anisopliae* and *Metarhizium acridum* also have large secretomes [4]. Many saprophytes have similarly sized secretomes as necrotrophs, as noted previously [2], which may reflect the fact that necrotrophs often have an extended saprophytic phase as part of their life cycle. Animal pathogens have fewer genes than saprophytes or plant-interacting fungi do, and a lower proportion of predicted secreted proteins.

## The Fungal Secretome Includes Carbohydrate-Degrading Enzymes and Effectors

Within the secretome there are different classes of proteins. Below we discuss two of them. Carbohydrate-degrading enzymes encoded by multigene families are secreted copiously by saprophytes to feed from complex molecules in the environment. Insect and plant pathogens that have to breach the host surface to gain entry also have large numbers, as do necrotrophs, which feed from tissue after they kill it. In contrast, biotrophs have few such families, as previously noted for mycorrhizae [5]; consequently, there is minimal release of pathogen-associated molecular patterns (PAMPs) from the plant cell wall. Accordingly, basal innate immunity, a mechanism common to animals and plants, is not triggered. Another class is effectors, which facilitate infection and/or induce defence responses [6]. Effectors are generally <300 amino acids, cysteine-rich, and lack transmembrane domains. They are often species-specific, polymorphic between isolates, and highly transcribed *in planta*. They can be avirulence proteins, which are complementary to plant resistance proteins in “gene for gene” interactions, host-specific toxins, or interfere with innate immunity by dampening or strengthening defence responses. Many proteins with effector-like properties have unknown functions.

## Effectors of Biotrophic Fungi Modulate Plant Responses

There are few genome sequences of biotrophic fungi, and as gene knockouts are difficult to carry out in such fungi, few effectors have been functionally analysed. Three that elicit plant responses have been characterized recently, two from symbionts and one from a pathogen. MISS7 from *L. bicolor* is the most highly upregulated gene during symbiosis with poplar. The encoded protein, which is crucial for successful symbiosis, moves to the nucleus where it modulates expression of poplar genes, including ones that alter root architecture [7]. A highly expressed effector from the mycorrhiza *Glomus intraradices*, SP7, moves to the plant nucleus where it interacts with pathogenesis-related transcription factor ERF19 and helps establish symbiosis, probably by dampening host defence [8]. A third effector that modulates plant responses is a chorismate mutase from *U. maydis*. This enzyme dimerises with a chorismate mutase from corn, and suppresses the production of salicylic acid, a key molecule in plant defence signaling [9].

## Effector Genes Can Move within and between Kingdoms

Effector genes are often located within repeat-rich regions, near telomeres, or even on lineage-specific chromosomes; as a result, these genes are readily lost, gained, or mutated [10]. The gene encoding the host-specific toxin ToxA of *Stagonospora nodorum* is located near a transposase and is present in another wheat pathogen, *Pyrenophora tritici-repentis* [11]. Transfer of *ToxA* from *S. nodorum* to *P. tritici-repentis* probably occurred by horizontal gene transfer (HGT) on co-infected wheat leaves about 50 years ago. Genes not only move between fungal genera, but also across kingdoms. Phylogenetic analyses have revealed transfer of effector genes from fungi to oomycetes with 8% of the secretome of the sudden oak death oomycete *Phytophthora ramorum* proposed to be derived by HGT from fungi [12].

## Animal Pathogens May Not Need Many Effectors

Generally, there are few barriers for a fungus to overcome when infecting animals. In many cases a fungus needs to be small enough to enter the host, survive at 37°C (in the case of mammalian pathogens), and evade immune responses [13]. Animal pathogens are often soil saprophytes that infect opportunistically, but, unlike most plant pathogens, some mammalian pathogens are not highly adapted to their hosts. Perhaps because of this, many animal fungal pathogens, in contrast to most plant fungal pathogens, do not display host specificity. An obvious exception to this is the insect pathogenic genus *Metarhizium*, which displays species specificity [4]. Furthermore, there is generally no intimate cellular relationship between fungus and animal as exists for obligate biotrophs or symbionts, which are enveloped in fungal and plant plasmalemmas. Thus, effectors may not be necessary to mediate deceit in all fungal–animal interactions, but are likely to be crucial for interactions that deceitful and affectionate fungi have with plants.

## References

[1] Singh RP, Hodson DP, Huerta-Espino J, Jin Y, Bhavani S (2011). The emergence of Ug99 races of the stem rust fungus is a threat to world wheat production.. Ann Rev Phytopathol.

[2] Choi J, Park J, Kim D, Jung K, Kang S (2010). Fungal Secretome Database: integrated platform for annotation of fungal secretomes.. BMC Genomics.

[3] Zuccaro A, Lahrmann U, Güldener U, Langen G, Pfiffi S (2011). Endophytic life strategies decoded by genome and transcriptome analyses of the mutualistic root symbiont *Piriformospora indica*.. PLoS Pathog.

[4] Gao Q, Jin K, Ying S-H, Zhang Y, Xiao G (2011). Genome sequencing and comparative transcriptomics of the model entomopathogenic fungi *Metarhizium anisopliae* and *M. acridum*.. PLoS Genet.

[5] Plett JM, Martin F (2011). Blurred boundaries: lifestyle lessons from ectomycorrhizal fungal genomes.. Trends Genet.

[6] De Wit PJ, Mehrabi R, Van den Burg HA, Stergiopoulos I (2009). Fungal effector proteins: past, present and future.. Mol Plant Pathol.

[7] Plett JM, Kemppainen M, Kale SD, Kohler A, Legue V (2011). A secreted effector protein of *Laccaria bicolor* is required for symbiosis development.. Curr Biol.

[8] Kloppholz S, Kuhn H, Requena N (2011). A secreted fungal effector of *Glomus intraradices* promotes symbiotic biotrophy.. Curr Biol.

[9] Djamei A, Schipper K, Rabe F, Ghosh A, Vincon V (2011). Metabolic priming by a secreted fungal effector.. Nature.

[10] Rep M, Kistler HC (2010). The genomic organization of plant pathogenicity in *Fusarium* species.. Curr Opin Plant Biol.

[11] Friesen TL, Stukenbrock EH, Liu Z, Meinhardt S, Ling H (2006). Emergence of a new disease as a result of interspecific virulence gene transfer.. Nat Genet.

[12] Richards TA, Soanes DM, Jones MDM, Vasieva O, Leonard G (2011). Horizontal gene transfer facilitated the evolution of plant parasitic mechanisms in the oomycetes.. Proc Nat Acad Sci U S A.

[13] Howlett BJ, Choffnes ER, Relman DA (2011). Fungal pathogenesis in plants and animals; similarities and differences.. Fungal diseases: an emerging challenge to human, animal and plant health.

[14] Rouxel T, Grandaubert J, Hane JK, Hoede C, van de Wouw AP (2011). Effector diversification within compartments of the *Leptosphaeria maculans* genome affected by Repeat-Induced Point mutations.. Nature Commun.

[15] Duplessis S, Cuomo CA, Lin Y-C, Aerts A, Tisserant E (2011). Obligate biotrophy features unraveled by the genomic analysis of rust fungi.. Proc Natl Acad Sci U S A.

[16] Martin F, Kohler A, Murat C, Balestrini R, Coutinho PM (2010). Perigord black truffle genome uncovers evolutionary origins and mechanisms of symbiosis.. Nature.

